# Post-Irradiation Facial Neuromyotonia/Myokymia: A Hemifacial Spasm Mimic

**DOI:** 10.5334/tohm.653

**Published:** 2021-09-20

**Authors:** Bart E. K. S. Swinnen, J. H. T. M. Koelman, A. F. van Rootselaar

**Affiliations:** 1 Department of Neurology and Clinical Neurophysiology, Amsterdam University Medical Centers, Amsterdam Neuroscience, University of Amsterdam, Amsterdam, The Netherlands

**Keywords:** hemifacial spasm, myokymia, neuromyotonia, irradiation, carbamazepine

## Abstract

**Background:**

Hemifacial spasm is diagnosed on a clinical base, with certain atypical features alerting the physician for mimics.

**Phenomenology shown:**

Hemifacial neuromyotonia/myokymia characterized by tonic hemifacial contraction followed by multifocal undulating hemifacial twitches.

**Educational value:**

These features are a red flag for (post-irradiation) facial neuromyotonia/myokymia which generally responds well to low dose carbamazepine.

## Main text

We present a 60-year-old person who had undergone gamma-knife surgery for right vestibular schwannoma at the age of 47 and 49. Since the age of 54 she experienced involuntary painful right unilateral facial contractions, frequency of which increased over time and occurred on average once daily at the moment of presentation. The contractions were elicited by facial movements (*e.g*. talking, yawning, and sneezing), facial thermal stimulation (*e.g*. facial hygiene, wind, and cold) and acidic food. They were characterized by tonic hemifacial contraction lasting one to two minutes, progressively transitioning into multifocal undulating hemifacial twitches slowly dissipating over several minutes (***Video 1***, part 1). Needle electromyography of frontal, orbicular ocular and mental muscles at rest revealed bursts of motor unit potentials consistent with myokymic discharges (***Video 1***, part 2). Tumor progression was excluded with magnetic resonance imaging at the age of 59. It was concluded that she suffered from facial neuromyotonia/myokymia due to post-irradiation facial nerve demyelination. Contribution of demyelination by previous (or current) facial nerve compression by the (residual) schwannoma could not be excluded. Whereas injections with botulinum toxin were unsuccessful, resolution of hemifacial contractions was obtained with carbamazepine 100 mg twice daily.

**Video 1:** **Right hemifacial neuromyotonia and myokymia**. *Part 1 (00:00 to 02:56)*; Home video obtained by the patient starting several seconds after onset of right hemifacial contraction. In the beginning of the video a tonic contraction (neuromyotonia) of the entire right facial musculature can be observed and lasts approximately one minute. This tonic contraction progressively transitions into undulating multifocal facial twitches (myokymia), dissipating over several minutes. *Part 2 (02:57 to 03:12)*; needle EMG of right mental muscle showing spontaneous bursts of single motor unit potentials with a frequency up to 100 Hz (sweep duration 500 ms, sensitivity 100 µV).

This case demonstrates facial neuromyotonia/myokymia to be a differential diagnosis of hemifacial spasm, next to facial tics, facial dystonia, facial myoclonus, facial synkinesis, functional neurological disorder, hemimasticatory spasm, epilepsy, and myorhythmia [1,2]. Neuromyotonia and myokymia constitute a continuum of peripheral nerve hyperexcitability clinically characterized by a tonic contraction and undulating rippling movements respectively [3]. The electrophysiological correlate of these are bursts of single motor unit potentials firing at 150 to 300 Hz and 5 to 150 Hz respectively. Red flags for facial neuromyotonia/myokymia are sustained tonic contractions, multifocal undulating rippling movements, myotonic or myokymic discharges on EMG, and history of radiotherapy or radiosurgery in the vicinity of the facial nerve. Although hemifacial spasm has a much higher incidence, recognition of facial neuromyotonia/myokymia is important as it has therapeutic and diagnostic implications. Neuromyotonia/myokymia generally responds well to low doses of sodium channel blockers (*e.g*. carbamazepine or phenytoin) as these decrease nerve hyperexcitability [3]. Post-irradiation neuromyotonia of other cranial nerves (*e.g*. oculomotor, trigeminal, spinal accessory, and hypoglossal nerves) has been described as well and is believed to be caused by delayed radiation-induced demyelination. Of interest, demyelination by tumor compression of the facial nerve (*e.g*. schwannoma) or intracerebral demyelination (*e.g*. multiple sclerosis) has been shown to equally induce facial neuromyotonia/myokymia [2]. Hence, brain imaging is warranted in such cases.
